# Ambient Electrochemical Ammonia Synthesis: From Theoretical Guidance to Catalyst Design

**DOI:** 10.1002/advs.202308979

**Published:** 2024-02-12

**Authors:** Jianjia Mu, Xuan‐Wen Gao, Tong Yu, Lu‐Kang Zhao, Wen‐Bin Luo, Huicong Yang, Zhao‐Meng Liu, Zhenhua Sun, Qin‐Fen Gu, Feng Li

**Affiliations:** ^1^ Institute for Energy Electrochemistry and Urban Mines Metallurgy School of Metallurgy Northeastern University Shenyang Liaoning 110819 China; ^2^ Institute of Metal Research Chinese Academy of Sciences Shenyang Liaoning 110016 China; ^3^ Australian Synchrotron (ANSTO) 800 Blackburn Rd Clayton VIC 3168 Australia

**Keywords:** electrocatalyst design, electrochemical ammonia synthesis, nitrate reduction reaction, nitrogen reduction reaction

## Abstract

Ammonia, a vital component in the synthesis of fertilizers, plastics, and explosives, is traditionally produced via the energy‐intensive and environmentally detrimental Haber–Bosch process. Given its considerable energy consumption and significant greenhouse gas emissions, there is a growing shift toward electrocatalytic ammonia synthesis as an eco‐friendly alternative. However, developing efficient electrocatalysts capable of achieving high selectivity, Faraday efficiency, and yield under ambient conditions remains a significant challenge. This review delves into the decades‐long research into electrocatalytic ammonia synthesis, highlighting the evolution of fundamental principles, theoretical descriptors, and reaction mechanisms. An in‐depth analysis of the nitrogen reduction reaction (NRR) and nitrate reduction reaction (NitRR) is provided, with a focus on their electrocatalysts. Additionally, the theories behind electrocatalyst design for ammonia synthesis are examined, including the Gibbs free energy approach, Sabatier principle, *d*‐band center theory, and orbital spin states. The review culminates in a comprehensive overview of the current challenges and prospective future directions in electrocatalyst development for NRR and NitRR, paving the way for more sustainable methods of ammonia production.

## Graphical abstract

This review delves into the decades‐long research into electrocatalytic ammonia synthesis, highlighting the evolution of fundamental principles, theoretical descriptors, and reaction mechanisms and paving the way for more sustainable methods of ammonia production.

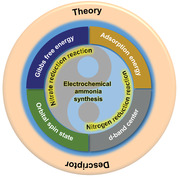

## 1 Introduction

Ammonia, a solution‐liquefaction product with a high hydrogen concentration, can be utilized as the fuel of energy carrier, which is of great importance for solving energy and environmental problems.^[^ ^1^, ^2^, ^3^, ^4^, ^5^ ^]^ As the society develops and the population increases, natural nitrogen reduction to ammonia based on microorganisms is far from meeting the growing needs of humans, the artificial ammonia production methods are necessary.^[^ ^3^, ^6^ ^]^ The Haber–Bosch method, a milestone in artificial ammonia synthesis, was developed in the early 20th century, in which high temperature (>300 °C) and intense pressure (>15 MPa) conditions are required due to the strong bond energies of the N≡N triple bond (≈941 kJ mol^−1^), and in particular the large energy barriers (410 kJ mol^−1^) to be overcome in the first step of N≡N triple bond dissociation.^[^ ^7^, ^8^, ^9^ ^]^ This process is thought to be responsible for 2% of the annual global total energy consumption and more than 1.9 tons of CO_2_ emission per ton of produced ammonia.^[^ ^3^, ^7^ ^]^ On the whole, conventional ammonia production through the Haber–Bosch process is environmentally damaging and non‐renewable. Therefore, there is an urgent need to explore an efficient artificial ammonia synthesis approach, preferably those with minimal infrastructure requirements and operational capabilities in ambient conditions and powered by clean renewable energy.^[^ ^6^, ^10^, ^11^ ^]^

Considering all the substitutes for Haber–Bosch method, the electrochemical ammonia synthesis is endowed with a potential ecologically acceptable process for sustaining artificial NH_3_ output under ambient conditions, which is thermodynamically driven through the adaptable electrochemical potentials.^[^ ^12^, ^13^, ^14^, ^15^ ^]^ Recently, the technology of electrochemical ammonia synthesis has gained a lot of interest due to its low energy consumption, environmental friendliness, and controlled reaction process, which has become an attractive field in electrocatalytic research. Regarding the nitrogen sources besides inert N_2_, nitrate, the highly active but ecologically hazardous nitrogen source, is considered a promising precursor to achieve efficient ammonia synthesis.^[^ ^16^, ^17^, ^18^, ^19^, ^20^, ^21^, ^22^, ^23^ ^]^ Although electrochemical ammonia synthesis has made significant advancements mainly benefiting from the integration of theoretical prediction and experimental investigations,^[^ ^12^, ^15^, ^24^, ^25^, ^26^, ^27^, ^28^, ^29^, ^30^, ^31^, ^32^, ^33^, ^34^ ^]^ developing and exploring extremely efficient electrocatalysts with high selectivity and stability remain certain challenges.

So far, several tactics have been devised to boost the performance of electrochemical ammonia synthesis, including defect engineering,^[^ ^35^, ^36^, ^37^ ^]^ interface engineering,^[^ ^38^, ^39^, ^40^, ^41^ ^]^ size effect,^[^ ^42^, ^43^ ^]^ alloying,^[^ ^44^, ^45^, ^46^ ^]^ and heteroatom doping^[^ ^47^, ^48^ ^]^ et al. The design of the aforementioned efficient electrocatalysts, however, still mainly relies on the conventional trial‐and‐error approach, which is frequently time‐consuming and ineffective. Fortunately, several theories and descriptors have emerged in the field of electrocatalysis over the years, including the Sabatier principle,^[^ ^49^ ^]^ *d*‐band center theory,^[^ ^28^ ^]^ and orbital spin states,^[^ ^42^ ^]^ among others. These conceptual frameworks have been instrumental in deepening our understanding the atomic‐level structure‐activity correlation and catalytic reaction mechanism in electrocatalysts. Furthermore, they have significantly expedited the developing high‐performance catalysts.

**Figure 1:**
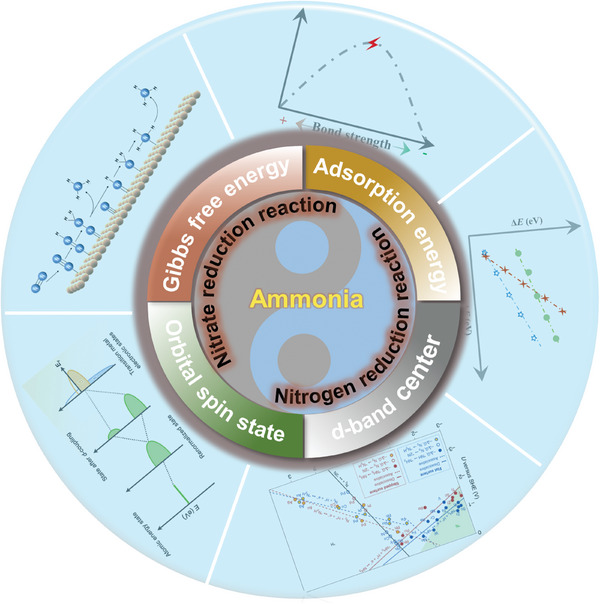
The possible electrochemical ammonia synthesis method and corresponding electrocatalytic theories.

In this review, we emphasized the fundamental understanding of electrochemical ammonia synthesis via the NRR and NitRR. By exploring the thermodynamics and kinetics mechanism as well as reaction pahthways, we aim to inspire new perspectives, encourage reflection, and spur further innovation in this field. Then, we summarize the previously reported electrocatalytic theories including Gibbs free energy, Sabatier principle, *d*‐band center theory, orbital spin state, etc., and especially concentrate on their application in electrocatalytic ammonia synthesis (**Figure** **1**). Subsequently, we comprehensively discuss the advanced electrocatalysts based on the abovementioned catalytic theories toward highly efficient ammonia synthesis. Ultimately, we conclude the major challenges and future outlooks for the theory‐guided electrocatalyst design toward electrochemical ammonia synthesis. We intend to comprehensively present the most recent developments in this subject, but more significantly, offer opportunities to address the issues confronting the advance of electrochemical ammonia production.

## 2 Mechanism of Electrochemical Ammonia Synthesis

For electrochemical ammonia synthesis, a great deal of theoretical understanding and practical expertise has been gathered over the last decades. With a few minor exceptions, the knowledge system of the thermodynamics and reaction processes of NRR and NitRR is generally well‐established. Unquestionably, a thorough understanding of these mechanisms is considerably advantageous for the construction of efficient NRR and NitRR electrocatalysts with abundant exposed active sites and high selectivity. The principles of NRR and NitRR are outlined in this section, with emphasis on several crucial procedures related to the properties of the electrocatalyst from the perspective of reactant and reaction pathway.

### 2.1 The Principles of NRR (Nitrogen Reduction Reaction)

#### 2.1.1 Reactant Properties

**Figure 2:**
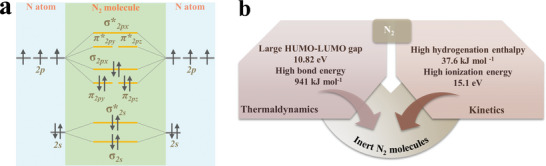
a) Simplified N_2_ molecular orbital diagram demonstrating the presence of both filled σ electrons and unfilled π^*^ orbitals in the N_2_ molecule. b) Illustration of inert N_2_ molecules according to the thermodynamic and kinetic properties.

Nitrogen molecule consists of two N atoms linked via a triple bond, each of which features an electronic configuration of 2s^2^2p^3^ in the outer orbital. When two N atoms combine to produce an N_2_ molecule, their electron clouds redistribute to generate eight fresh molecule orbitals through a linear combination of atomic orbitals from each N atom (1 × s and 3 × p), including four bonding orbitals (2 × *σ* and 2 × *π*) and four anti‐bonding orbitals (2 × σ^*^ and 2 × π^*^). The ten valence electrons occupy four bonding orbitals and one anti‐bonding orbital (σ^*^_2s_). As a result, the highest occupied molecular orbital (HOMO) and the lowest unoccupied orbital (LUMO) of N_2_ molecules are one *σ* orbital and two degenerate π* orbitals, respectively (**Figure** **2**a). Therefore, the N≡N triple bond can be effectively weakened by donating their paired *σ*‐electrons to the unoccupied orbital of the electrocatalyst, or accepting electrons from the electrocatalyst into their unoccupied *π* ^*^ orbital, or synchronizing both processes. From thermodynamic and kinetic standpoints, the activation of N_2_ molecules is challenging owing to the following three reasons (Figure 2b). 1) The effective electron transfer is severely hampered by the energy gap of 10.82 eV between the LUMO and HOMO of N_2_ molecules.^[^ ^13^ ^]^ 2) The cleavage energy of the N≡N triple bond in N_2_ molecules reaches 941 kJ mol^−1^, which is almost a thermodynamically infeasible process.^[^ ^50^ ^]^ 3) The hydrogenation enthalpy for N_2_ molecules in the first protonation step is positive (+37.6 kJ mol^−1^) and the ionization energy is ≈15.1 eV, indicating it is a thermodynamically forbidden reaction.^[^ ^51^ ^]^ Notably, although the first protonation process (N_2_ + H^+^→N_2_H^+^) is endothermic, the following hydrogenation step is thermodynamically favored, resulting in a relatively low theoretical potential of 0.092 V versus RHE.^[^ ^50^ ^]^

#### 2.1.2 Possible Reaction Routes of NRR

Gaining insight into the electrochemical nitrogen synthesis is made possible by the natural biological reduction of N_2_ to NH_3_ on nitrogenases under ambient circumstances. Just inspired by this process, numerous electrocatalysts were created and the associated reaction mechanism was proposed. Generally, the typical route of electrochemical ammonia synthesis in an aqueous electrolyte relates to four crucial processes, including N_2_ adsorption on the active sites, activation of inert N≡N triple bond through electron or H^+^, proton–electron transfer step, and NH_3_ desorption on the surface of electrocatalysts.

**Figure 3:**
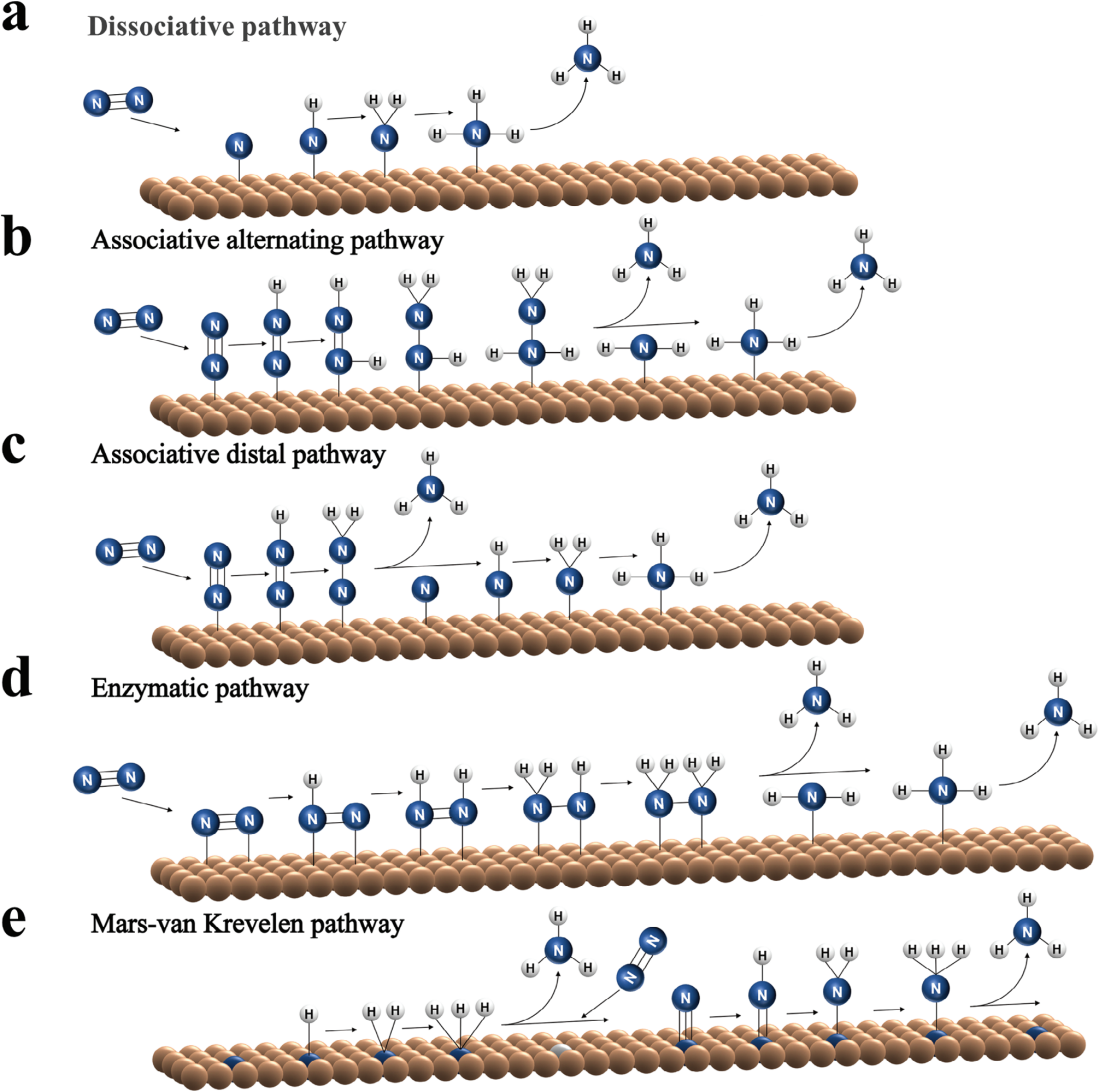
Schematic illustration of the nitrogen reduction reaction routes.

The theoretical investigation has identified several potential reaction routes on the active center for the electrocatalytic N_2_ reduction to NH_3_, containing the dissociative pathway, the associative pathway, the enzymatic pathway, and the Mars‐van Krevelen (MvK) pathway, as shown in **Figure** **3**. In the dissociative pathway, the cleavage of the N≡N triple bond happens before protonation, in which case a large energy input is required to thoroughly broken N≡N triple bond^[^ ^50^, ^52^ ^]^ (Figure 3a). Generally, the Haber‐Bosch process follows the dissociative mechanism under high temperature and pressure. Unlike the dissociative pathway, the cleavage of the N≡N triple bond is accomplished through successive protonations in the associative pathway,^[^ ^53^ ^]^ accompanied by ammonia release, which prevails in the electrochemical nitrogen reduction process due to its gentle reaction conditions. According to the different hydrogenation processes, the associative pathway is divided into the distal pathway, the alternative pathway, and the enzymatic pathway as shown in Figure 3b,c. Specifically, in the distal pathway, only one N atom adsorbs on the active center of electrocatalysts, and the other one away from the electrocatalyst is preferentially protonated to produce the first ammonia molecule. The N≡N triple bond completely breaks with the emission of the first ammonia molecule, the remaining N atom adsorbed on the active center continues to be attacked by protons and electrons to form the second ammonia molecule. Different from the distal pathway, in the alternative hydrogenation pathway, the two N atoms are alternately hydrogenated to release two ammonia molecules at the same time. In an enzymatic way, the two N atoms chemically adsorbed on the active sites of electrocatalysts on a side‐on bonding model followed by an alternate or simultaneous proton‐coupled electron process^[^ ^51^, ^54^ ^]^ (Figure 3d). Finally, a reaction mechanism involving lattice N has been proposed during NRR by metal nitrides, in which the lattice N from electrocatalysts is reduced to NH_3_ through combining the protons, leaving a nitrogen vacancy.^[^ ^55^ ^]^ Then, the gaseous N_2_ molecules will interact with the nitrogen vacancy to reproduce lattice N. The details for this mechanism are presented in Figure 3e.

Electrochemical NRR is a multi‐step process involving six electrons, where the reaction kinetics is slow and a hydrogen evolution competition reaction is prone to occur. In contrast, the hydrogen evolution reaction (HER) is a two‐electron reaction process, which seriously hinders the NRR reaction process through occupying the active centers. Generally, a definite reaction pathway for NRR on the active center can be influenced by multiple factors, including the types of electrocatalysts, applied potentials, the local environment of the reaction interface, etc. Without employing theory simulations, a specific NRR reaction pathway in a particular electrocatalytic system can not be established due to the synergistic influence of multiple variables. Moreover, it is crucial to comprehend these processes based on the thoroughly theoretical and experimental investigations for developing and exploring the highly efficient and selective NRR electrocatalysts.

### 2.2 The Principles of NitRR (Nitrate Reduction Reaction)

Compared with NRR, ambient electrochemical conversion of nitrate to ammonia features its intrinsic advantages. Nitrate ions with high reactivity are more easily reduced, which not only reduces energy consumption but also lowers the cost of operation and equipment. Moreover, when nitrate ions are employed as the reactant, the high selectivity toward ammonia facilitates the overall energy efficiency. Electrocatalytic nitrate reduction to ammonia in aqueous electrolyte, a complex proton‐coupled electron transfer process involving multiple intermediates and products, has become a promising substitute for NRR. In this process, nitrate‐containing wastewater as a nitrogen source is electrochemically converted into ammonia, which is a green “waste‐to‐treasure” method for electrochemical ammonia synthesis.

**Figure 4:**
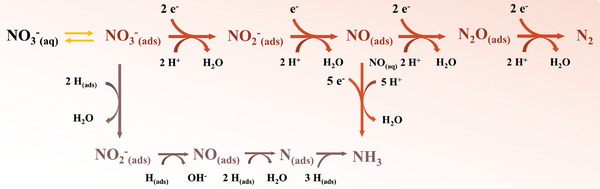
Schematic illustration of the direct nitrate reduction reaction routes.

Generally, electrocatalytic nitrate reduction follows two mechanisms: the indirect autocatalytic^[^ ^56^, ^57^ ^]^ and the direct electro‐reduction pathway.^[^ ^58^, ^59^ ^]^ In the typical indirect autocatalytic mechanism, nitrate itself does not engage in the electron transfer process. Instead, intermediates like NO^+^ or NO_2_ participate in this process. Consequently, this reaction mechanism predominantly occurs in acidic electrolytes containing high‐concentration of nitrate. The direct electroreduction of nitrate involves two pathways (**Figure** **4**), namely electron‐mediated nitrate reduction and actively adsorbed hydrogen (*H*_ads_) reduction. According to the electron‐mediated nitrate reduction, the initial step corresponds to the conversion of adsorbed NO_3_^−^_(ads)_ to NO_2_^−^_(ads)_, followed by the reduction of NO_2_^−^_(ads)_ to NO_(ads)_. N_2_ or NH_3_ may be the ultimate reductive products due to thermodynamic stability. Besides, the atomically adsorbed hydrogen can function as a strong reductive agent (E_(H+/H)_ = −2.31 V versus SHE) to reduce the adsorbed nitrate and other intermediate species.^[^ ^60^ ^]^ Notably, from a kinetic point of view, the migration energy barrier of *H*_ads_ (0.1 eV) is much lower than that of *N*_ads_ (0.75 eV), the formation of N─H bonds is a kinetically supported process compared with N─N bonds.^[^ ^61^, ^62^ ^]^

### 2.3 Theory and Descriptors of Electrocatalyst Design

For the complex electrochemical ammonia synthesis (six‐electron NRR and eight‐electron NitRR), theoretical guidance plays an important role not only in understanding the reaction mechanisms but also in designing efficient electrocatalysts. After extensive theoretical calculations and experimental validation, several theories and descriptors for the design of efficient electrocatalysts have been proposed. In this section, we systematically summarize the reported theories, descriptors and assess their feasibility for application in ammonia synthesis electrocatalyst design. Specifically, Gibbs free energy is a parameter that describes the thermodynamic properties of the electrocatalytic system, through which researchers propose the electrocatalytic reaction process on the surface of electrocatalysts can be affected by the adsorption energy of reaction intermediates. According to the Sabatier principle, the optimal electrocatalysts should have proper adsorption energy for the reactants and intermediates in the electrocatalytic process. Furthermore, the *d*‐band theory quantitatively understands the adsorption behavior between catalytic materials and intermediates from an electronic perspective, through which the active centers can be regulated to boost the intrinsic electrocatalytic activity. The spin state from crystal field theory further refines the d orbital of electrocatalysts, which is associated with the adsorption intensity of intermediates on the electrocatalyst surface. Thus, combining the theory and descriptors, the active centers can be identified and regulated to enhance the electrochemical performance in the electrocatalytic reaction.

#### 2.3.1 Gibbs Free Energy

Computational hydrogen electrode model has been proposed by Norskov et al.,^[^ ^63^ ^]^ with which the Gibbs free energy (*ΔG*) of every elementary reaction can be obtained during electrocatalytic ammonia production. Specifically, the Gibbs free energy was calculated based on the following Equations 1 and 2

(1) $$\Delta G=\Delta E+\Delta \mathrm{ZPE}-T\Delta S+\Delta G_{U}$$

(2) $$\Delta E=E_{T}-E_{\mathrm{catalyst}}-E_{\mathrm{adsorbate}}$$

Where Δ*E*, the adsorption energy of reactant N_2_ and corresponding intermediates, is calculated through density functional theory (DFT), Δ*ZPE* corresponds to the changes in the zero point energy, TΔ*S* is assigned to alternation in entropy at the set temperature, Δ*G*_U_ represents the influence of the electrode potential (U) on Δ*G*, *E*_T_ is systematically total energy during electrochemical ammonia synthesis, *E*_catalyst_ is the free energy of electrocatalyst, and *E*_adsorbate_ corresponds to the free energy of adsorbed reactant. From a thermodynamic standpoint, the elementary reaction with the maximum free energy (Δ*G*_max_) is potential‐determining step. The reaction routes and electrocatalyst activity can be evaluated by comparing Δ*G*_max_. Through the Gibbs free energy of intermediates,

#### 2.3.2 Sabatier Principle

**Figure 5:**
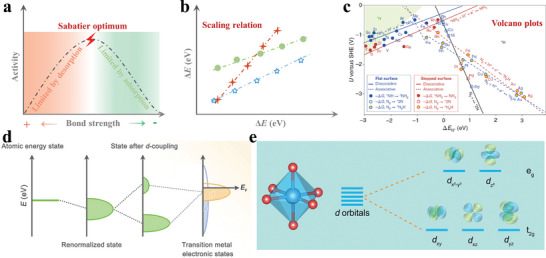
a) Schematic diagram of the Sabatier principle. b) Scaling relationship between various intermediates during NRR process. c) Volcano curve of adsorbed N^*^ on the surface of electrocatalysts: the relationship between applied potential and the binding energy of adsorbed N^*^. Reproduced with permission.^[^ ^69^ ^]^ Copyright 2012, Royal Society of Chemistry. d) Schematic illustration of chemical interaction between the reaction intermediates and the *s/d*‐band from electrocatalysts. Reproduced with permission. Reproduced with permission.^[^ ^71^ ^]^ Copyright 2022, Elsevier. e) The formation process of *e*_g_ and *t*_2g_ via *d* orbital splitting in an octahedral field. Reproduced with permission. Reproduced with permission.^[^ ^72^ ^]^ Copyright 2023, Wiley VCH.

The aforementioned analysis reveals that the adsorption energy of the intermediates on the electrocatalysts significantly impacts the reaction process. Sabatier principle, a general theory for heterogeneous electrocatalysts, demonstrates that the ideal electrocatalysts should feature the proper adsorption energy to the reactants and intermediates during the electrocatalytic process.^[^ ^64^, ^65^ ^]^ Too strong adsorption prevents the desorption of reaction intermediates and products, and too weak adsorption is detrimental to the chemical adsorption of reactants, both of which would enable slow kinetics of the electrocatalytic reaction (**Figure** **5**a). Furthermore, according to the Brønsted‐Evans‐Polanyi principle, the adsorption energy of intermediates to electrocatalysts is linearly related to another for a multiple‐step complicated electrocatalytic reaction,^[^ ^66^, ^67^ ^]^ as shown in Figure 5b. Therefore, the Sabatier principle can be simplified by the adsorption energy of the key intermediate as an activity descriptor. The volcano plots were built to explore potentially novel electrocatalysts for electrochemical ammonia synthesis (Figure 5c), in which the applied potential for NRR and HER is characterized as a function of the ΔE_N*_ (the adsorption energy of N atom to electrocatalysts).^[^ ^68^, ^69^ ^]^ The adsorption energy of N_2_H^*^ can also serve as an activity descriptor for NRR. By combining it with the activity descriptor of HER, the selectivity of the NRR can be evaluated based on the free energy difference between H^*^ and N_2_H^*^ (Δ*G*_H*_ − Δ*G*_N2H*_). When Δ*G*_H*_ − Δ*G*_N2H*_ > 0, it indicates that the catalyst is favorable for N_2_^*^ hydrogenation, thus demonstrating good selectivity.^[^ ^70^ ^]^

#### 2.3.3 *d*‐Band Theory

From an orbital perspective, the adsorption of adsorbates onto electrocatalysts surfaces can be viewed as the coupling of the adsorbates’ orbitals with the orbitals of the electrocatalysts. Occupying high‐energy anti‐bonding states by electrons will decrease overall stability, which is unfavorable for adsorption. In transition metals, *s* and *p* orbitals exhibit broad overlapping shape, while *d* orbitals are localized (Figure 5d). It is generally accepted that the coupling between adsorbates' orbitals and *s* or *p* orbitals of different transition metals is similar. Therefore, *d*‐band electronics states directly influence the adsorption behavior.^[^ ^73^ ^]^ This is the renowned *d*‐band theory, which has been extensively employed for the prediction of electrocatalytic activity of catalysts.^[^ ^74^ ^]^ The *d*‐band center is the simplest descriptor for *d*‐band theory and is used to describe *d*‐band electrons. The stronger bonding between electrocatalysts and adsorbates originates from the higher anti‐bonding energy level (less readily occupied by electrons), which is correlated with the higher *d*‐band center (closer to the Fermi energy level). Moreover, the *d*‐band width,^[^ ^75^ ^]^ *d*‐orbital charge,^[^ ^76^ ^]^ and *p*‐band center for nonmetal materials^[^ ^77^ ^]^ have also been proposed as descriptors to the adsorption states.

#### 2.3.4 Spin State

The descriptors proposed based on *d*‐band theory primarily serve to describe the average state of *d*‐orbital electrons and have certain limitations when applied to magnetic materials with a significant number of unpaired electrons, such as transition metal oxides.^[^ ^72^ ^]^ Therefore, it is imperative to consider the influence of electron spin state, and further refine the electronic state of the *d* orbital. In ligand field theory, transition metals are subject to influences from ligands (nonmetal in compounds or adsorbates), resulting in a splitting of their *d* orbitals.^[^ ^78^ ^]^ Take the octahedral structure as an example, the *d*‐electrons closer to the ligand feature larger energies than those farther away. Therefore, the *d* orbitals will split into two groups with various energy levels including the low‐energy *t*_2g_ orbitals (*d*_xy_, *d*_xz_, and *d*_yz_) and high‐energy *e*_g_ orbitals (*d_z_^2^* and *d_x_^2^_‐y_^2^*), as shown in Figure 5e. Affected by the splitting energy and the electron pairing energy, the electrons may exhibit high spin, intermediate spin, and low spin states. The spin state difference has an impact on charge transfer and adsorption behavior. For instance, high spin states tend to generate more unpaired electrons, leading to enhanced electrical conductivity. The electrocatalyst performs exceptionally well catalytically and has the most suitable adsorption capability for important intermediates when the electron filling number of the *e*_g_ orbit is ≈1.^[^ ^72^ ^]^

## 3 Progress of Electrocatalyst Based on the Theory and Descriptors

The electrocatalysts, the most crucial component for efficient electrochemical ammonia synthesis, have been studied consistently, containing carbon materials, alloy, and metal oxides/sulfides/nitrides/phosphides/carbides, etc.^[^ ^79^ ^]^ Various strategies^[^ ^51^, ^79^ ^]^ were also explored to design the adsorption site with inherent catalytic activity and high selectivity toward ammonia production instead of protons, such as defect engineering, vacancy engineering, alloying, interface engineering, size effect, electronic metal‐support interaction, etc. The aforementioned electrocatalysts and their corresponding strategies mainly depend on tedious trial‐and‐error methods. In the following section, we primarily summarize the previously reported catalysts toward efficient NRR and NitRR based on the above theory and descriptors, in order to theoretically guide the synthesis of high‐efficiency electrocatalysts toward high ammonia yield rate and Faradic efficiency. Additionally, the electrochemical performance of partial electrocatalysts was sorted and provided in **Table** **1**.

**Table 1:** Recently reported results on electrochemical NRR.

Materials	Electrolyte	Yield rate / µg h^−1^ mg_cat._^−1^	FE	Refs.
Zn^1^N‐C	0.1 _M_ KOH	16.10	11.80%	[15]
Mo_2_C/C	0.5 _M_ Li_2_SO_4_	11.30	7.80%	[80]
FeNi_2_S_4_/NiS	0.1 _M_ KOH	128.4	28.60%	[81]
1T‐MoS_2_	0.1 _M_ Na_2_SO_4_	71.07	21.01%	[26]
Fe‐SnO_2_	0.1 _M_ HCl	82.70	20.40%	[27]
Mo_2_C‐MoO_2_	0.1 _M_ Na_2_SO_4_	13.94	12.72%	[28]
V‐doped TiO_2_	0.5 _M_ LiClO_4_	17.73	15.30%	[29]
FeTPPCl	0.1 _M_ Na_2_SO_4_	18.28	16.76%	[30]
Bi_2_S_3−x_/Ti_3_C_2_T_x_	0.1 _M_ KOH	68.30	22.50%	[40]
MoS_2_/C_3_N_4_	0.1 _M_ LiClO_4_	18.50	17.80%	[41]
Rh@CNT	0.1 _M_ PBS	26.91	23.48%	[42]
Ti_3_C_2_T_X_	0.1 m HCl	36.90	9.10%	[43]
W_18_O_49_‐16Fe	0.25 _M_ LiClO_4_	24.70	20.00%	[48]
TiO_2−δ_N_δ_	0.1 _M_ KOH	14.33	9.17%	[82]
FeMo dimer	0.1 _M_ LiClO_4_	14.95	41.70%	[83]
D‐FeN/C	0.1 _M_ KOH	24.80	15.80%	[84]

### 3.1 NRR Electrocatalysis

#### 3.1.1 The Gibbs Free Energy for NRR

Recently, theoretical calculations based on DFT have become a general approach for predicting reaction pathways and screening reaction intermediates of electrocatalytic reactions. In this process, the Gibbs free energy (Δ*G*) of each elementary reaction can be determined through Equation 1, which can reflect the electrocatalytic activity of catalysts. Abundant theoretical and experimental research has presented that many strategies, including heteroatom doping,^[^ ^47^, ^48^, ^82^ ^]^ single atom,^[^ ^83^ ^]^ and alloying^[^ ^44^, ^45^, ^46^ ^]^ et al., have a positive influence on the regulation of the Gibbs free energy for NRR.

**Figure 6:**
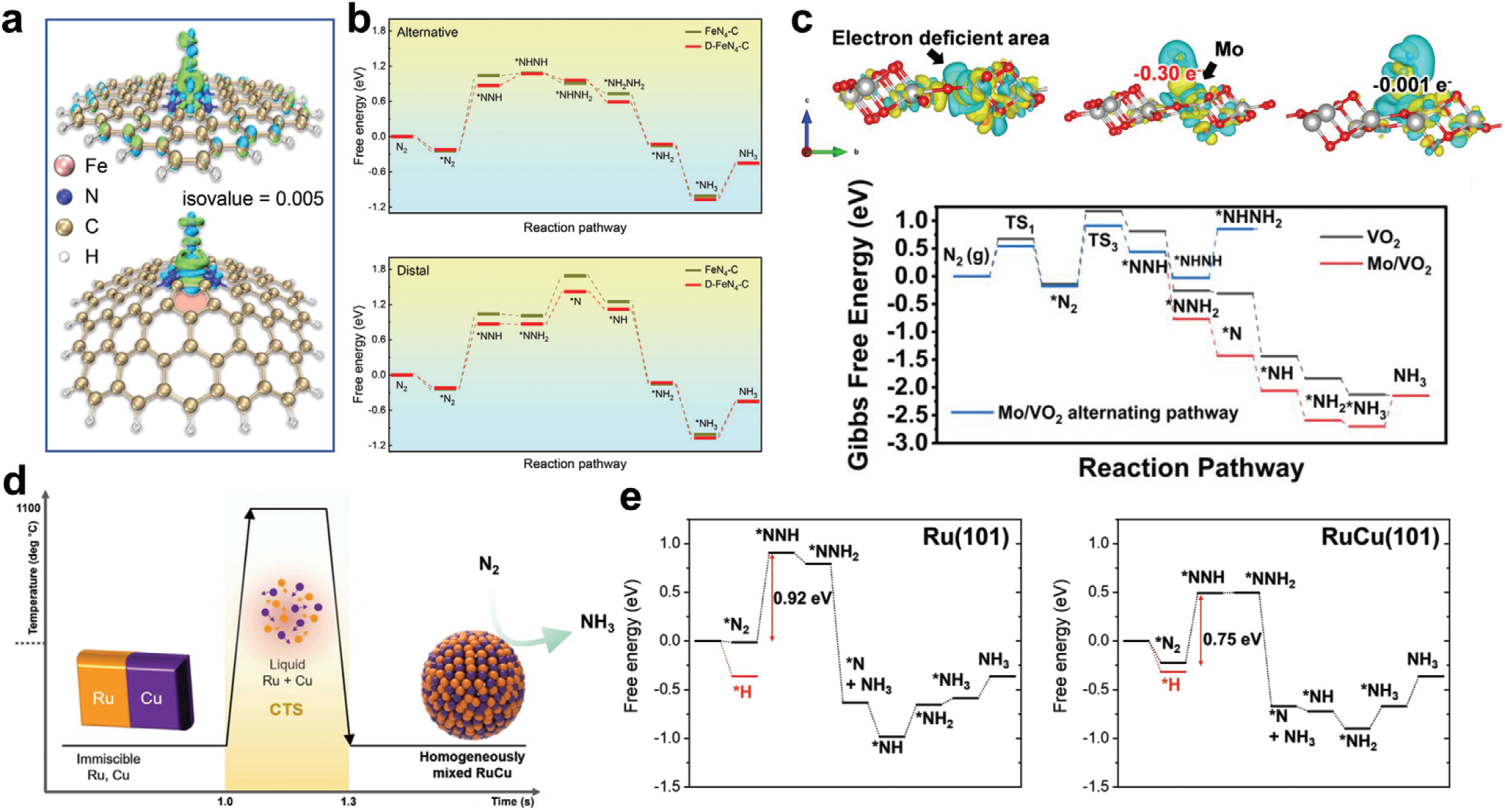
a) The difference of electron density of ^*^N_2_ intermediates adsorbed on FeN_4_‐C and D‐FeN_4_‐C models, and b) corresponding free energy diagram based on the alternative and distal pathway. Reproduced with permission.^[^ ^84^ ^]^ Copyright 2022, Wiley VCH. c) The charge density of charge density difference of Mo/VO_2_, Mo/VO_2_ after N_2_ adsorption, VO_2_ for N_2_ adsorption, and Gibbs free energy diagrams for NRR. Spheres: red = O, gray = V, cyan = Mo. Reproduced with permission.^[^ ^85^ ^]^ Copyright 2023, Wiley VCH. d) Schematic illustration of the synthesis of Ru–Cu nanoparticles. e) Free energy diagram of Ru and RuCu for NRR. Reproduced with permission.^[^ ^86^ ^]^ Copyright 2022, Wiley VCH.

As shown in **Figure** **6**a, atomically dispersed FeN_4_ anchored in porous nitrogen‐doped defect‐rich carbon nanofibers (D‐FeN/C) was constructed via combined single atom with defect engineering, which enabled an efficient charge transfer from Fe site to adsorbed ^*^N_2_ through backdonation mechanism and elongate the bond length of the N≡N triple bond to 1.12 Å. Compared with FeN/C, D‐FeN/C featured an energy barrier of 1.32 eV for the generation of H^*^, which was beneficial to the first protonation step of ^*^N_2_ (Figure 6a). Additionally, the free energy diagram presented that the introduction of carbon defects can significantly reduce the energy barrier of the first protonation step in both the associative alternative pathway and the associative distal pathway (Figure 6b). As a result, the alkaline NRR performance of D‐FeN/C exhibited a considerably high ammonia yield rate of 24.8 µg h^−1^ mg_cat._^−1^ and Faradic efficiency of 15.8% at −0.4 V versus RHE, better than nearly all known Fe‐based NRR catalysts.^[^ ^84^ ^]^ In addition to the Fe elements, the Mo elements have received a lot of attention in the NRR process. The effect of electronic metal‐support interaction between metal Mo and VO_2_ on NRR performance was evaluated (Figure 6c), in which the electron‐defect‐rich Mo induced via electronic metal‐support interaction can effectively destabilize ^*^N_2_ and lower the energy barrier of the subsequent hydrogenation process.^[^ ^85^ ^]^ Compared with VO_2_, the enhanced yield rate (10.8 times) and Faradic efficiency (2.8 times) were obtained by the addition of Mo. The ∆*G*(^*^N_2_) − ∆*G*(^*^H) was chosen as the descriptor of NRR selectivity shown in Figure 6d,e,^[^ ^86^ ^]^ where RuCu with more negative value (∆*G*(^*^N_2_) − ∆*G*(^*^H)) achieved a higher Faradic efficiency compared with that of Ru. Besides, homogeneously mixed bimetallic RuCu nanoparticles with more negative free energy of ^*^N_2_ is a high‐efficiency NRR electrocatalysts with an ammonia yield rate of 73 µmol h^−1^ cm^−2^ and Faradic efficiency of 31%. DFT results indicated the alloying facilitates the N_2_ adsorption and the following hydrogenation process (Figure 6e).

#### 3.1.2 Adsorption Energy for NRR

**Figure 7:**
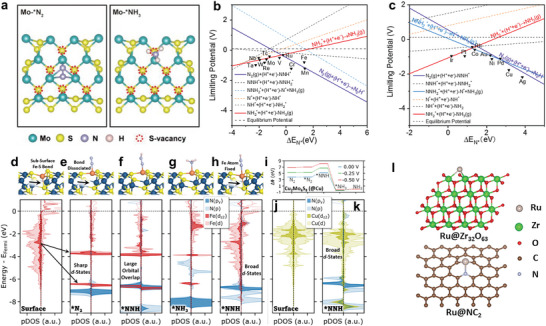
a) Schematic diagram of adsorbed N_2_ and NH_3_ on Mo sites surrounded by defects. Reproduced with permission.^[^ ^88^ ^]^ Copyright 2023, Wiley VCH. Relationship between limiting potential and adsorption energy of N^*^ for single‐atom catalysts. b) For the early transition metals and c) for the last transition metals. Reproduced with permission.^[^ ^89^ ^]^ Copyright 2019, American Chemical Society. Structure model and projected density of states (PDOS) for d) the pristine Fe_2_Mo_6_S_8_ surface, the Fe_2_Mo_6_S_8_ surface with e) ^*^N_2_, f) ^*^NNH, g) ^*^NH_3_, h) ^*^NNH with the Fe fixed at its position on the clean surface. i) The free energy diagram on the Cu_2_Mo_6_S_8_ surface and corresponding PDOS of j) clean surface and k) ^*^NNH adsorbed on the surface. Reproduced with permission.^[^ ^92^ ^]^ Copyright 2022, American Chemical Society. l) Structure model of Ru@Zr_32_O_63_ and Ru/NC_2_. Reproduced with permission.^[^ ^93^ ^]^ Copyright 2019, Elsevier.

According to the Sabatier principle, the electrocatalytic nitrogen reduction process is significantly dependent on the adsorption energies of the reactants and intermediates. Specifically, when ^*^N strongly adsorbs on the surface of electrocatalysts, the comprehensive activity is limited by ^*^NH_2_ → NH_3_, that is, the strong bonding of intermediates deteriorates the desorption of products. When the chemisorption capacity of ^*^N on the active center of electrocatalysts is inadequate, the first hydrogenation of ^*^N_2_ (^*^N_2_ → ^*^N_2_H) is restricted, that is, the weak adsorption of ^*^N does not favor to initial NRR process. The electrocatalytic activity of catalysts toward NRR can be well described by the ^*^N adsorption energy, which is a descriptor for constructing efficient electrocatalysts.^[^ ^68^ ^]^ Benefiting from recent theoretical and experimental studies, several strategies have been extensively proposed and explored, such as defect engineering,^[^ ^35^, ^36^, ^37^ ^]^ edge effect,^[^ ^87^ ^]^ and alloying,^[^ ^44^, ^45^, ^46^ ^]^ etc. As presented in **Figure** **7**a, the bonding capacity of N_2_ on MoS_2_ can be enhanced due to the high electron density and strong electron localization around Mo atoms surrounded by a certain concentration of S‐vacancy, achieving an excellent electrocatalytic performance including an ammonia yield rate of 66.74 µg h^−1^ mg_cat._^−1^ and Faradic efficiency of 14.68%.^[^ ^88^ ^]^ In addition to vacancy engineering, the edge effect of VS_2_ is extremely advantageous to boost the adsorption capacity of N_2_ through the “acceptance‐donation” mechanisms.^[^ ^87^ ^]^ Single‐atom materials have been chosen as the model catalysts to evaluate the relationship between electrocatalytic activity and ΔE_N*_. Through the DFT calculations, 60 kinds of atomically dispersed transition metal anchored on N‐doped carbon supports as NRR electrocatalysts was evaluated, presenting a strong correlation between the intrinsically electrocatalytic activity and the adsorption energy of N^*^ (Figure 7b,c). Additionally, the bonding/antibonding orbital populations should take responsibility for the as‐obtained trends of ΔE_N*_ on the active center of TM‐SACs.^[^ ^89^ ^]^ This work suggests a general engineering strategy to explore efficient NRR catalysts with tunable electronic structure.

In theelectrocatalytic NRR, both reactants and N‐containing intermediates are adsorbed onto the active sites of the catalysts through metal‐N bond. This leads to a certain correlation between the adsorption energies of various reaction intermediates. Consequently, there is a notable relationship between the catalytic activity and ΔE_N*_, which typically follows a volcano‐like trend. Notably, the scaling relationship between NRR reaction intermediates greatly limits the activity of NRR electrocatalysts, leading to a low Faradic efficiency. Therefore, how to break the scaling relations has been a hot topic, which is advantageous for designing efficient NRR electrocatalysts. The reported strategies include bifunctionality, promoters, tethering/functionalization, electrolyte engineering, interface sites, and confinement effect, etc.^[^ ^90^, ^91^ ^]^ Besides, according to the theoretical and experimental investigation,^[^ ^71^ ^]^ the metal nitrides, such as ZrN, VN, and NbN, may be an ideal electrocatalyst to remove the limitation of scaling relations. Generally, NRR process with the metal nitrides as the electrocatalyst follows the MvK mechanism, during which each elementary reaction could become the rate‐limiting step. Besides the metal nitrides, some other efforts also provide a possibility for breaking the scaling relations during electrocatalysis. Through the grand‐canonical DFT, a novel NRR mechanism on Fe_2_Mo_6_S_8_ surface was proposed to get rid of the limitation of scaling relations.^[^ ^92^ ^]^ Specifically, the active Fe sites on Fe_2_Mo_6_S_8_ preferentially stabilize the key ^*^NNH intermediates instead of ^*^N_2_ through the narrow band of d‐states formed by the cleavage of Fe─S bond after N_2_ adsorption, which enables the Fe sites to break the scaling relation during nitrogen electrocatalysis (Figure 7d–k).

Besides the scaling relations, the volcano plots also can efficiently guide the design of electrocatalysts toward NRR. As shown in Figure 5c, the studies have provided an overview of transition metals, containing Ru, Au, Fe, Rh, Mo, Sc, Ti, Y, and Zr, with appropriate ΔE_N*_. Recently, a large number of electrocatalysts based on Sc, Ti, Y, and Zr elements have been reported, because they locate at the dominant region of N_2_ adsorption in the volcano plot, indicating their intrinsic HER inertness.^[^ ^68^ ^]^ Our group synthesized N‐doped TiO_2_ nanowire arrays on carbon cloth (TiO_2‐δ_N_δ_@CC), achieving an ammonia production rate of 14.33 µg h^−1^ mg_cat._^−1^ and Faradic efficiency of 9.17%. More importantly, the introduction of heteroatom N can effectively lower the adsorption energy of protons, which facilitates a high Faradic efficiency.^[^ ^82^ ^]^ The effect of support (Ru@Zr_32_O_63_ and Ru@NC_2_ electrocatalysts) was evaluated during NRR process, and the results indicated that the introduction of ZrO_2_ can effectively boost the Faradic efficiency to 21% by suppressing the binding of ^*^H on the active center^[^ ^93^ ^]^ (Figure 7l).

#### 3.1.3 *d*‐Band Center for NRR

The location of the *d*‐band center and its migration are important parameters for evaluating the electrocatalytic activity of the catalysts. Since electrocatalytic reactions typically occur at the surface of catalysts, the electrocatalytic activity can be modulated by optimizing the *d*‐band center of the catalyst surface, closely related to the adsorption energy and activation energy barrier of the reaction intermediates during the electrocatalytic process. The closer the *d*‐band center is to the Fermi level, the stronger the bond capability between the electrocatalysts and reactive intermediate.

**Figure 8:**
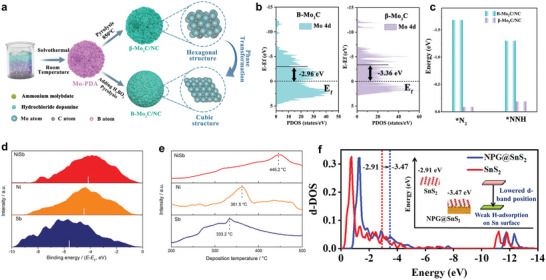
a) Schematic diagram of the synthesis process of B‐Mo_2_C/NC and β‐Mo_2_C/NC. b) The projected density of states (PDOS) of Mo 4d from B‐Mo_2_C/NC and *β*‐Mo_2_C/NC catalysts. c) The Gibbs free energy change of adsorbing N_2_ and forming *NNH on the active center of B‐Mo_2_C/NC and β‐Mo_2_C/NC. Reproduced with permission.^[^ ^94^ ^]^ Copyright 2022, Wiley VCH. d) Surface valence of NiSb, Ni, and Sb measured from XPS. e) The N_2_ temperature‐programmed desorption behavior of NiSb, Ni, and Sb. Reproduced with permission.^[^ ^95^ ^]^ Copyright 2021, Wiley VCH. f) The regulation of the *d*‐band center of Sn on the SnS_2_ surface. Reproduced with permission.^[^ ^97^ ^]^ Copyright 2021, American Chemical Society.

As shown in **Figure** **8**a, B‐doping strategy was introduced into the preparation procedure of β‐Mo_2_C/NC to synthesize B‐Mo_2_C/NC characteristic of optimal electronic states and surface properties. After doping, the *d*‐band center of Mo 4*d* from B‐Mo_2_C/NC moved to the Fermi energy, indicating a stronger interaction between electrocatalyst and ^*^N_2_ species, which facilitated lowering the NRR energy barrier and stabilizing the intermediates (Figure 8b). Further, Figure 8c presented the Gibbs free energy changes (ΔG_*N2_ andΔG_*NNH_) of the formation of ^*^N_2_ and ^*^NNH, which could significantly reduce the ΔG_*N2_ from −0.086 to −1.68 eV and the ΔG_*NNH_ from −0.187 to −1.3 eV, respectively, enhancing the adsorption of ^*^N_2_ and ^*^NNH on B‐Mo_2_C/NC. Generally, the surface electronic effect can be described by XPS or UPS valence band spectra, which can link its center with the filling of the antibonding states of adsorbates.^[^ ^94^ ^]^ As shown in Figure 8d, the bimetallic NiSb exhibited an upshift of valence band spectra approaching the Fermi level compared with Ni and Sb, implying the stronger bonding strength of intermediates due to less‐filled antibonding states of adsorbents, which was in line with the results of N_2_ temperature‐programmed desorption profiles^[^ ^95^ ^]^ (Figure 8e). By coupling Pd element to Fe, the valence band center of Fe was regulated and the adsorption of reactants was promoted by our group.^[^ ^96^ ^]^ On the other hand, it is also possible to reduce *d*‐band center of the active sites to impair the corresponding interaction with intermediates. As shown in Figure 8f, the downshift of the d‐band center from NPG@SnS_2_ is beneficial for suppressing the adsorption of *H, which can enhance the Faradic efficiency from 5.2% to 37.1% through suppressing HER.^[^ ^97^ ^]^

#### 3.1.4 Orbital Spin for NRR

**Figure 9:**
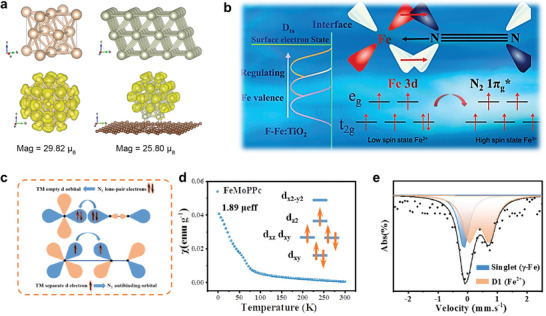
a) The spin‐resolved density diagrams of bulk Rh (top left), corresponding (111) plane of bulk Rh (top right), Rh cluster (down left), and Rh cluster/graphene (down right). Reproduced with permission.^[^ ^42^ ^]^ Copyright 2021, Elsevier. b) The schematic illustration of high‐spin state Fe doped TiO_2_. Reproduced with permission.^[^ ^98^ ^]^ Copyright 2022, Elsevier. c) Schematic illustration of the interaction between N_2_ and transition metals. Reproduced with permission.^[^ ^99^ ^]^ Copyright 2021, Wiley VCH. d) Magnetic susceptibility and (e) Ambient ^57^Fe Mössbauer spectrum of FeMoPPc. Reproduced with permission.^[^ ^99^ ^]^ Copyright 2021, Wiley VCH.

The orbital spin state of catalysts has a significant impact on their electrocatalytic activity, which can influence the adsorption behavior of reactants and intermediates through regulating the electron transfer and reaction energy barrier. As shown in **Figure** **9**a, Rh clusters supported on graphene (Rh@G) were synthesized with large spin magnetic moment of 39.82 µ_B_. DFT results indicated the presence of a high‐spin electron density at the edge/surface of Rh cluster, which promoted the N_2_ adsorption and activation process, achieving a high ammonia yield rate of 26.91 µg h^−1^ mg_cat._^−1^ and Faradic efficiency of 23.48%.^[^ ^42^ ^]^ In addition to the size effect, the regulation of the valence state of Fe also can obtain high‐spin Fe‐doped TiO_2_ catalyst for efficient NRR (Figure 9b), indicating that the spin state can enable Fe 3*d* electrons to backdonate to antibond of N_2_ to lower the limiting potentials during NRR process.^[^ ^98^ ^]^ Previous researches have revealed a strong correlation between catalytic activity and the electronic configuration of the transition‐metal *e*_g_ orbitals. The catalyst exhibits optimal adsorption strength with the critical reaction intermediates, resulting in excellent electrocatalytic performance, when the number of electron fillings in the *e*_g_ orbitals is close to one.^[^ ^72^ ^]^ For instance, due to the complex coordination environments, the electrons feature various distributions in d orbitals of transition metals including low spin state (Fe^3+^: *t*_2g_^5^e_g_^0^ and Co^3+^: *t*_2g_^6^e_g_^0^), intermediate spin state (Fe^3+^: *t*_2g_^4^ *e*_g_^1^ and Co^3+^: *t*_2g_^5^ *e*_g_^1^), and high spin state (Fe^3+^: *t*_2g_^3^ *e*_g_^2^ and Co^3+^: *t*_2g_^4^ *e*_g_^2^). As shown in Figure 9c–e, the orbital spin states are quantitatively evaluated by theoretical calculations and experimental verification.^[^ ^99^ ^]^ Fe and Mo single atoms with a FeN_4_ and MoN_4_ configuration were simultaneously constructed in a polyphthalocyanine (PPc) organic framework to evaluate the positive effect of the spin state of FeN_4_ on NRR. The transformation of the spin states of FeN_4_ from the high‐spin state (*d_xy_^2^d_yz_^1^d_xz_^1^d_z_^21^d_x_^2^_‐y_^21^*) to medium‐spin (*d_xy_^2^d_yz_^1^d_xz_^1^d_z_^21^*) can be achieved by the neighboring MoN_4_, in which Fe *3d* and N *2p* orbitals can effectively overlap, indicating the strong interaction between electrocatalyst and N_2_. Theoretical calculations show that the transition of the Fe spin state reduces the energy barrier for the determination step and favors the first protonation step.

### 3.2 NitRR Electrocatalysis

Electrocatalytic nitrate reduction to ammonia under ambient conditions offers an eco‐friendly method for treating NO_3_^−^/NO_2_^−^‐containing wastewater and is a viable alternative to NRR due to its high selectivity and fast reaction kinetics.^[^ ^59^, ^100^, ^101^, ^102^, ^103^, ^104^, ^105^, ^106^, ^107^, ^108^ ^]^ Moreover, the practical experience in NRR can be applied to NitRR process, and the abovementioned theory and descriptors also can guide the design of NitRR catalysts.

**Figure 10:**
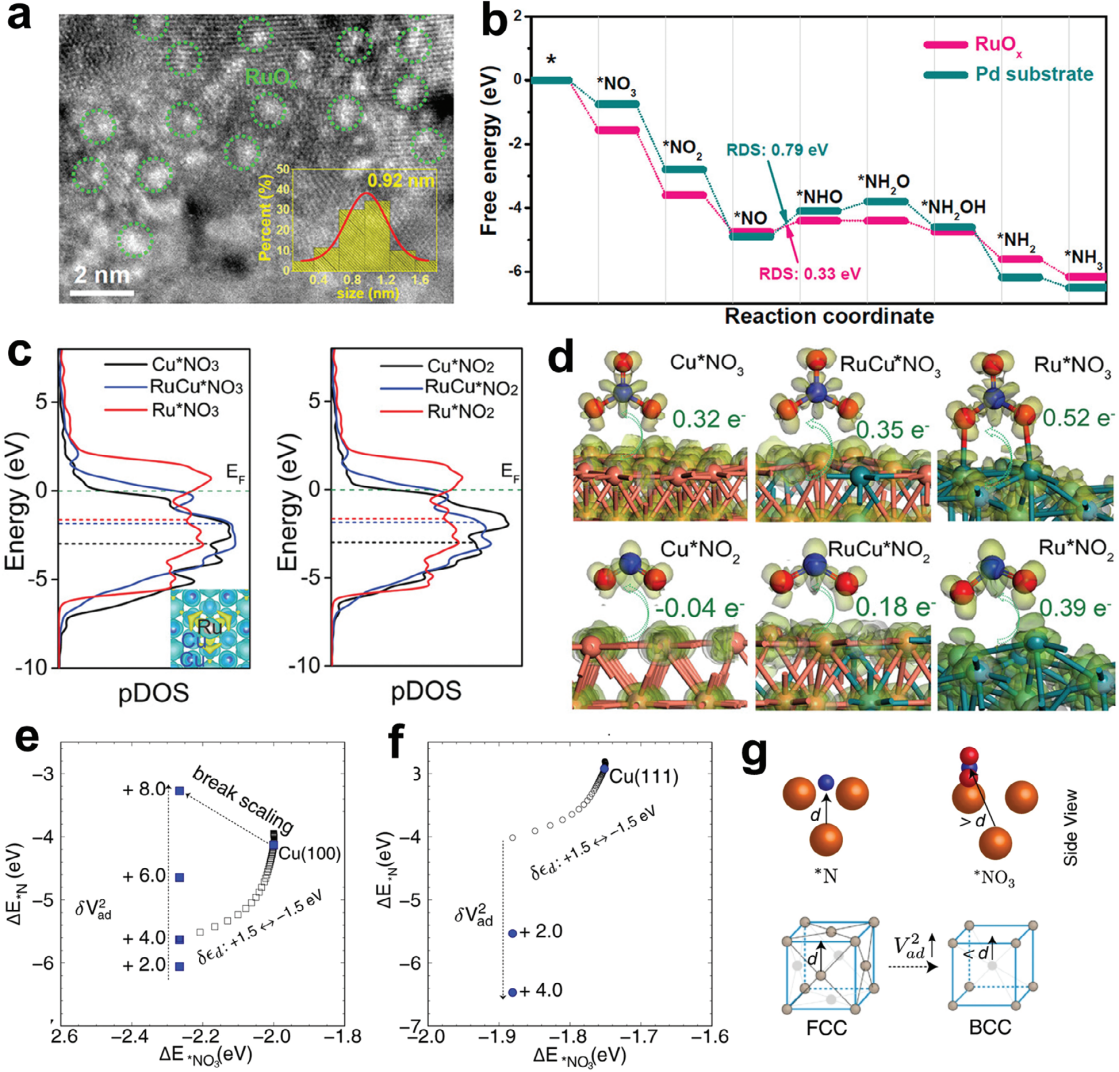
a) HADDF‐STEM image of RuO_x_/Pd. b) Free energy diagram of NitRR on Pd and RuO_x_/Pd. Reproduced with permission.^[^ ^109^ ^]^ Copyright 2023, American Chemical Society. c) Project density of states of Cu, Ru, and RuCu with NO_3_^−^ and NO_2_^−^ adsorption. Dash lines correspond to *d*‐band center. Reproduced with permission.^[^ ^110^ ^]^ Copyright 2023, Wiley VCH. d) Charge density differences and electron transfer of Cu, Ru, and RuCu with NO_3_^−^ and NO_2_^−^ adsorption. The adsorption energies of ^*^NO_3_^−^ and ^*^N on e) Cu(110), f) Cu(111). g) Schematic illustration for breaking scaling relation between ^*^NO_3_^−^ and ^*^N. Reproduced with permission.^[^ ^111^ ^]^ Copyright 2022, Springer Nature.

Sub‐nm RuO_x_ clusters supported on a Pd metallene were synthesized with a high ammonia production rate of 23.5 mg h^−1^cm^−2^ and a Faradic efficiency of 98.6% (**Figure** **10**a,b). DFT results showed that the synergistic effect between RuO_x_ clusters and Pd metallene is beneficial for lowing the adsorption energy of ^*^NO_3_^−^, in which the dissociation of H_2_O to ^*^H was achieved by Pd, and RuO_x_ clusters were responsible for ^*^NO_3_^−^ adsorption and activation.^[^ ^109^ ^]^ Additional DFT results indicated that the ^*^H on Pd sites gradually migrate toward the RuO_x_/Pd interface to participate into the nitrate reduction reaction, indicating thermaldynamically supported ^*^H spillover process. The *d*‐band center also has a positive effect on designing efficient NitRR catalysts. As shown in Figure 10c,d, the alloying between Ru and Cu could enable the *d*‐band center of active centers to upshift toward the Fermi level compared with Cu, which is advantageous to accelerate the charge transfer.^[^ ^110^ ^]^ Bader charge results also indicated that RuCu with optimized *d*‐band center can effectively activate ^*^NO_3_^−^ via charge transfer. In addition, the synergistic effect of Ru and Cu sites was systematically investigated, where Ru presented better activity for converting NO_2_^−^ to NH_3_ and Cu displayed exceptional activity for reducing NO_3_^−^ to NO_2_^−^, comprehensively resulting in a satisfactory ammonia formation rate of 0.38 mmol cm^−2^ h^−1^ and Faradic efficiency of 98% at −0.05 V versus RHE. Similar to the NRR process, the scaling relations between the adsorption energies of intermediates exist during NitRR. With the assistance of machine learning (Figure 10e,f), a strategy to break scaling relations via the site‐specific Pauli repulsion interactions of the metal d‐states was proposed^[^ ^111^ ^]^ (Figure 10g).

## 4 Conclusions and Perspectives

**Figure 11:**
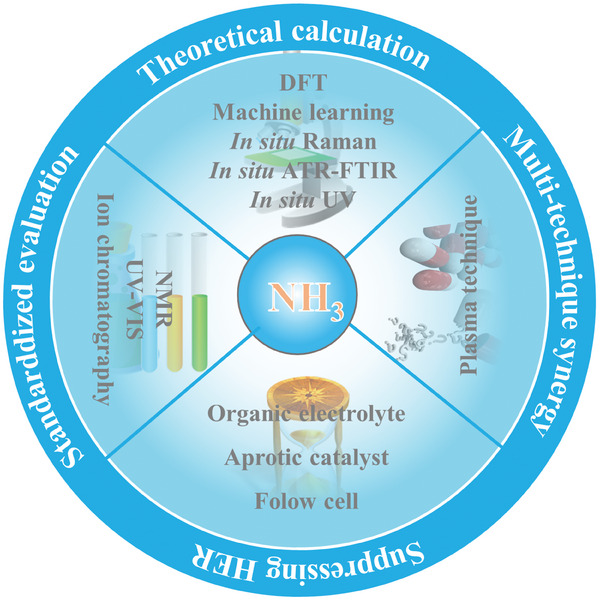
A schematic representation of the perspectives toward electrochemical ammonia synthesis.

Despite significant advances in recent years, electrochemical ammonia synthesis from nitrogen or nitrate still faces hurdles in commercialization and industrialization, primarily due to its suboptimal intrinsic activity and limited selectivity. Hence, a thorough understanding of the electrocatalytic mechanisms involved in the NRR and NitRR is crucial. We delve into various electrocatalytic theories pertinent to NRR and NitRR, including Gibbs free energy, the Sabatier principle, *d*‐band center theory, and orbital spin states. Furthermore, we explore the emerging opportunities and challenges in the realm of electrochemical ammonia synthesis, aiming to provide insights into future research directions as shown in **Figure** **11**.

### 4.1 Theoretical Calculation Combined with In Situ Characterization

We have synthesized insights from several theories and descriptors (including Gibbs free energy, Sabatier principle, *d*‐band center, and orbital spin state) and their applications in electrochemical ammonia synthesis. While these frameworks explain catalytic activity post‐experimentation, they often fall short in accurately predicting complex electrochemical reactions due to their reliance on simplistic approximations and idealized structures. Thus, we need to build more integrated descriptors to overcome the shortcomings of an individual descriptor, such as low prediction accuracy and a limited application scope for a specific electrocatalytic reaction. One critical aspect in electrochemical ammonia synthesis is the role of electrode potential on electrocatalysts. This potential influences electron occupancy and spin states, which in turn affect adsorption behavior and reactivity. Notably, it has been observed that electrode potential can reverse adsorption energy trends.^[^ ^112^ ^]^ Additionally, NRR and NitRR typically occur in aqueous electrolytes under reduction potentials lower than 0 V versus SHE, a condition that may lead to the reconstruction of electrochemically active species. Given these complexities, advanced in situ techniques are paramount for monitoring real‐time dynamic changes in both geometric and electronic structures of catalysts and intermediates. These techniques include in situ FTIR spectroscopy, in situ UV–vis spectroscopy, in situ mass spectrometry, and in situ Raman spectroscopy, among others. Machine learning and theoretical computational approaches can then be utilized to decipher patterns from the collected data, aiding in the identification of optimal descriptors for evaluating electrocatalyst activity. Therefore, an integrated approach that combines theoretical predictions (DFT and machine learning techniques) with experimental characterization data emerges as a powerful tool in identifying reliable theory descriptors. This synergy can significantly guide the design of next‐generation electrocatalysts for ammonia synthesis.

### 4.2 Multi‐Technique Synergistic Nitrogen Fixation

One such innovative method involves a two‐step process for converting N_2_ to ammonia. This method begins with plasma pre‐processing to oxidize N_2_ into more soluble NO_x_^−^, followed by an electrocatalysis process that efficiently converts NO_x_^−^ to NH_3_ with high selectivity.^[^ ^113^, ^114^ ^]^ This plasma technique thus addresses the solubility challenge by initially converting N_2_ to a more reactive and soluble form. In addition to artificial plasma methods, nitrogen can also be converted into nitrate through natural chemical and electrochemical reactions under certain extreme conditions. For instance, UV radiation can cause N_2_ and O_2_ in the air to react, forming active chemicals like NO and NO_2_. These substances subsequently interact with other atmospheric molecules to produce nitrate. Therefore, integrating various complementary approaches, including plasma processing and natural conversion processes, may offer a promising pathway for achieving efficient conversion of N_2_ to NH_3_.

### 4.3 Suppressing HER Activity

In liquid electrolytes, the two‐electron HER kinetically outpaces the six‐electron NRR in proton‐coupled electron transfer processes. This dominance leads to exceedingly low Faradaic efficiency and poor NRR selectivity. Consequently, devising effective strategies to suppress the competitive HER side reactions is imperative. Future research directions should include: 1) Developing organic or low‐proton‐activity electrolytes for NRR: Minimizing proton accessibility can significantly reduce hydrogen evolution. This can be achieved through alkali metal cation‐mediated electrocatalytic systems and high pH electrolytes, which offer lower proton availability. 2) Exploring high‐performance electrocatalysts with N affinity: certain early transition metals (such as Sc, Ti, Zr, and Y) have shown intrinsic affinity toward nitrogen rather than protons. Investigating these materials could lead to electrocatalysts that preferentially bind to nitrogen, thereby repelling protons and enhancing NRR selectivity. 3) Designing flow cell reactors with gas‐diffusion electrodes: implementing a gas‐diffusion electrode can substantially reduce the diffusion distance of N_2_, improving the efficiency of the NRR process. These approaches aim to address the current limitations in NRR selectivity and efficiency, presenting promising avenues for future research in the field.

### 4.4 Standardized Performance Evaluation

The assessment of electrochemical ammonia synthesis performance has focused on yield rate, Faradic efficiency, and stability. However, a significant challenge arises from the widespread presence of ammonia‐containing contaminants in laboratory settings. These contaminants, found in materials like electrocatalysts, carbon paper, Nafion solution, Nafion films, and electrolytes, can skew measured ammonia levels. To ensure the reliability of data, developing a comprehensive and accurate quantitative analysis method for electrocatalytic NH_3_ synthesis is imperative. While spectroscopic methods are commonly used for ammonia detection due to their practicality, relying solely on them can lead to data inconsistencies. Therefore, a multi‐technique approach, incorporating methods such as ion chromatography and liquid nuclear magnetic resonance (NMR), is essential to validate trace ammonia yields and eliminate uncertainties. Furthermore, to foster transparency and repeatability in this field, researchers should publish open‐access original data, particularly electrochemical data. Detailed experimental procedures must be thoroughly documented to enable replication and contribute robustly to the advancement of ammonia synthesis research.

The U.S. Department of Energy has announced technical indicators for commercial electrochemical ammonia synthesis in 2016, including current density >300 mA cm^−2^, Faradic efficiency >90%, energy efficiency >60%, and service lifespan >1000 h. Currently, the performance of electrochemical NRR from N_2_ is far from meeting the commercial standard, thus a thorough investigation of the ammonia synthesis system is required, including electrocatalyst engineering (high activity, selectivity, and stability), electrolyte optimization (high Faradic efficiency), and electrocatalytic reaction cell design (high current density toward N_2_ electroreduction).^[^ ^115^ ^]^ Besides NRR, the electrochemical reduction of nitrate is an alternative approach for commercial ammonia production with extensive nitrogen‐containing industrial sewage, which significantly improves the ammonia yield rate and Faradic efficiency by transforming the gas‐liquid‐solid three‐phase reaction into a liquid‐solid two‐phase reaction.

In conclusion, our review synthesizes several theoretical descriptors from recent work aimed at enhancing electrocatalysts for NRR and NitRR under ambient conditions. This burgeoning field is poised for significant breakthroughs that could revolutionize industrial ammonia production methods.

## Conflict of Interest

The authors declare no conflict of interest.
